# Double-blind, placebo-controlled randomized trial with adalimumab for treatment of juvenile onset ankylosing spondylitis (JoAS): significant short term improvement

**DOI:** 10.1186/ar4072

**Published:** 2012-10-24

**Authors:** Gerd Horneff, Sigrid Fitter, Ivan Foeldvari, Kirsten Minden, Jasmin Kuemmerle-Deschner, Nicolay Tzaribacev, Angelika Thon, Michael Borte, Gerd Ganser, Rolf Trauzeddel, Hans-Iko Huppertz

**Affiliations:** 1 General Pediatrics, Asklepios Clinics, Arnold Janssen Str. 29, Sankt Augustin, 53757, Germany; 2 Hamburger Zentrum für Kinder- und Jugendrheumatologie, Klinikum Eilbek, Dehnhaide 120, Hamburg 22081, Germany; 3 German Rheumatism Research Centre, Charitéplatz 1, Berlin, 10117, Germany; 4 Division of Pediatric Rheumatology, University Hospital Tübingen, Hoppe-Seyler-Straße 1, Tuebingen, 72076, Germany; 5 Department für Kinderrheumatologie, Klinikum Bad Bramstedt, Oskar-Alexander-Straße 26, Bad Bramstedt, 24576, Germany; 6 Department of Pediatric Pneumology, Allergology and Neonatology, Hannover Medical School Carl-Neuberg-Straße 1, Hannover, 30625, Germany; 7 Department for Pediatric Rheumatology, Klinikum St. Georg, Delitzscher Straße 141, Leipzig, 04129, Germany; 8 Department of Paediatric Rheumatology, St. Josef-Stift Sendenhorst, Westtor 7 Sendenhors, 48324, Germany; 9 4Klinik für Kinder- und Jugendmedizin, Helios Klinikum Berlin-Buch, Schwanebecker Chaussee 50, Berlin, 13125, Germany; 10 Department of Pediatrics, Prof. Hess-Kinderklinik, St.-Jürgen-Straße 1, Bremen, 28177, Germany

## Abstract

**Introduction:**

While adalimumab is licensed for ankylosing spondylitis (AS), open uncontrolled studies suggest therapeutic efficacy of TNF-inhibitors in juvenile onset AS (JoAS).

**Methods:**

A total of 32 patients aged 12 to 17 years with severe, active and refractory JoAS were enrolled in a multicenter, randomized, double-blind, placebo-controlled parallel study of 12 weeks, followed by open-label adalimumab until week 24 for all patients. ASAS40 was used as the primary, and ASAS20, PedACR and single items were used as the secondary outcome measures for the intention to treat population.

**Results:**

A total of 17 patients were randomized to receive adalimumab 40 mg/2 weeks and 15 patients received placebo. Two patients (one of each group) discontinued prematurely due to insufficient efficacy and were labeled as non-responders. In the double-blind part, more patients on adalimumab achieved an ASAS40 at week 4 (41%), week 8 (53%) and week 12 (53%) than on placebo (20%, 33%, 33%), while differences at week 8 only reached borderline significance (*P *= 0.05). Also, at 4, 8 and 12 weeks ASAS20/PedACR30/70 response rates were higher in the adalimumab group (53%/53%/29%; 59%/76%/41%; 53%/65%/53%) compared to placebo (27%/27%/7%; 27%/33%/13%; 33%/40%/27%). In the adalimumab group a significant decrease of all disease activity parameters was noted at week 12 and was even more pronounced at week 24. At week 12 the Bath Ankylosing Spondylitis Disease activity spinal inflammation score decreased by 65% (*P *<0.001), the back pain score decreased by 50% (*P *<0.005), the Bath AS Functional Index (BASFI) score decreased by 47% (*P *<0.02), while the Childhood Health Assessment Questionnaire-Disability Index (CHAQ-DI) score improved by 65% (*P *<0.005). ANCOVA analysis demonstrated superiority of adalimumab over placebo for the physician global assessment of disease activity, parents' global assessment of subject's overall well-being, active joint count (all *P *<0.05) and erythrocyte sedimentation rate (ESR) (*P *<0.01).

During the 12-week controlled phase, 29 AEs occurred in 10 patients on placebo compared to 27 AEs in 11 patients on adalimumab. Injection site reactions were the most common adverse events. There were 17 various infections occurring in the double-blind phase, 8 on placebo, 9 on adalimumab and a further 19 in the open label period.

**Conclusions:**

Adalimumab was well tolerated and highly effective in a double-blind randomized trial in patients with JoAS. Treatment effects rapidly occurred and persisted for at least 24 weeks of treatment.

**Trial registration:**

EudraCT 2007-003358-27.

## Introduction

Ankylosing spondylitis (AS) is a chronic inflammatory rheumatic disease that affects 0.2 to 0.8% of the population [1]. Although AS typically presents in the early 20s, it can present in childhood. In juvenile onset AS (JoAS), manifestations start in individuals <16 years of age and progress to sacroiliitis and spine involvement later on. Among patients with AS, prevalence rates for juvenile-onset vary from 9% to 21% in white populations [2].

Juvenile- and adult-onset spondyloarthropathies, particularly AS, differ in several aspects. Most differences consist of symptoms at the onset [3-7]. Adults are more likely to present with axial manifestations. In contrast to adults, children and adolescents with JoAS have peripheral arthritis and enthesitis in the initial years and axial symptoms 5 to 10 years later. The severity of AS is greater in juveniles than in adults since more juveniles require hip replacements, are in functional classes III and IV, and exhibit higher mean Bath AS Functional Index (BASFI) scores.

Differences in functional outcome have also been reported that depend on the age of onset. In a study comparing 24 JoAS with 71 adult AS patients, JoAS had worse functional outcome [8]. Early-course JoAS is often remitting. The number of peripheral joints involved remains limited with hips, knees, ankles and feet affected. Persistent peripheral joint involvement may be more frequent in JoAS than in adult AS and, particularly coxitis, may lead to a worse outcome.

JoAS describes a disease of childhood and adolescents which is not incorporated in juvenile idiopathic arthritis (JIA) [9]. The enthesitis and arthritis category of the juvenile idiopathic arthritis covers patients with exclusively peripheral joint involvement and those with additional axial involvement [10]. Therefore, most of the patients with JoAS will probably fulfill the diagnosis of the enthesitis and arthritis category of the JIA classification [10].

So far, treatment options are limited for JoAS. Nonsteroidal anti-inflammatory agents (NSAIDs) are the mainstay of treatment providing symptomatic relief. Disease modifying drugs (DMARDs) like methotrexate and other immunosuppressants have not shown to be useful for treatment of JoAS. Systemic and intra-articular corticosteroids promote susceptibility to infections, osteoporosis and growth disturbance.

There is now accumulating evidence that anti-TNF therapy is highly effective in adult AS [11-13]. There are five tumor necrosis factor alpha (TNFα)-blockers currently available: adalimumab, certolizumab, etanercept, golimumab and infliximab. Adalimumab is the first fully human monoclonal antibody engineered by gene technology that uses site-directed mutagenesis to enhance its binding efficiency to TNF. It does not contain non-human or artificial protein sequences [14]. Adalimumab binds only to TNFα [15] and has a half-life of approximately two weeks [16]. The antibody has been extensively studied *in vitro *as well as *in vivo *and is non-toxic in animal toxicology studies. Adalimumab has been studied for treatment of active resistant polyarticular JIA [17]. Severe adverse events have remained rare but infections, including tuberculosis, have been reported [18-20]. Currently, the benefits of anti-TNF therapy seem to outweigh these shortcomings.

The first evidence of therapeutic efficacy of TNF-inhibitors in patients with juvenile spondylarthopathies, including JoAS, was published years ago as case series or open studies [21-24].

Adalimumab so far has not been studied in JoAS patients but has been in adult AS. Those studies resulted in the approval of TNF inhibitors for adult AS, while in children and adolescents, TNF inhibitors are licensed only for polyarticular juvenile rheumatoid arthritis/juvenile idiopathic arthritis as well as inflammatory bowel disease [25-29].

In view of these results, we chose to study adalimumab in children with JoAS. For this study, only those patients were selected who had active axial involvement of JoAS with both clinical (limitation of movement of axial pain) and magnetic resonance imaging (MRI) evidence.

## Materials and methods

### Patients

In the absence of validated diagnostic criteria for JoAS, diagnosis of JoAS in this study required the following two conditions (I) bilateral active sacroiliitis confirmed by magnetic resonance imaging OR unilateral active sacroiliitis confirmed by magnetic resonance imaging and active peripheral joint disease restricted to the lower extremities (hip, knee, ankle) AND (II) at least one of three clinical criteria: (A) limitation of lumbar spine motion in all three planes, (B) pain or history of pain at the dorsolumbar junction of the spine and/or (C) limitation of chest expansion to 2.5 cm or less at the level of the fourth intercostal space.

Patients included were at least 12 and up to 17 years of age with a weight of at least 30 kg. Manifestations were refractory to two different non-steroidal anti-inflammatory drugs given at appropriate dosage and for a total of four weeks.

Active disease was defined by a spinal inflammation score of at least three (see below) AND at least two of the following criteria: (1) back pain score of at least 3; (2) patient global assessment of disease activity of at least 3; (3) physical function score as determined by the BASFI of at least 3.

Patients had to have stable doses of NSAIDs. Low doses of corticosteroids of no more than 0.2 mg of prednisone per kilogram body weight per day, with a maximum of 10 mg per day, were allowed. Intra-articular and soft-tissue corticosteroid injections were not permitted for four weeks prior to the Screening Visit. Patients treated with etanercept or infliximab or adalimumab or anakinra at any time for any period or with antimalarials, gold salts, sulfsalazine, azathioprine, penicillamine, leflunomide, cyclosporine A, intravenous immunoglobulin or methotrexate within four weeks prior to the first administration of study medication, or with plans to begin the intake of these drugs were excluded. Additional major exclusion criteria were a history of any chronic disease other than JoAS, JRA/JIA, especially chronic renal disease, liver disease, hematological, gastrointestinal, pulmonary, cardiological or neurological disease, which in the opinion of the investigator might influence the efficacy or safety of the study medication or which in the opinion of the investigator might lead to an unacceptable risk for the patient.

### Study medication and dosage

On the basis of pharmacokinetic considerations and recent experience with dosing of adalimumab in children, a dose of 40 mg every other week was given. The study drug was provided as an injection solution in prefilled syringes containing 0.8 ml of placebo or adalimumab 50 mg/ml concentration. Stable doses of NSAIDs and low dose of corticosteroids (≤10 mg per day) were permitted throughout the study period.

### Study design

This was a multi-center, randomized, double-blind, parallel-group Phase III study. Patients with JoAS received 1:1 40 mg of adalimumab or placebo subcutaneously every other week for a 12-week period (Controlled Phase). Clinical assessments were carried out at baseline and after 4, 8 and 12 weeks. At week 12 all patients who finalized the 12-week double-blind study received adalimumab. Further study visits occurred after 16, 20 and 24 weeks.

### Adverse events

Clinical and laboratory evidence of adverse events on a routine basis were completed throughout the study. The investigator assessed and recorded any adverse event in detail on the adverse event form, including the date and time of onset, description, severity, time course, duration and outcome, relationship of the adverse event to study drug and alternative etiology for events not considered 'probably related' to study drug.

### Efficacy evaluations

The clinical response to adalimumab was assessed using the Assessment of SpondyloArthritis International Society (ASAS) [30,31], the pediatric ACR (PedACR) criteria [32], the Bath Ankylosing Spondylitis Disease activity score (BASDAI) and the Bath Ankylosing Spondylitis Functional Index (BASFI) [33]. The individual domains contributing to the ASAS are (i) the spinal inflammation, defined as mean of items 5 (overall level of morning stiffness) and 6 (duration of morning stiffness) of the BASDAI; (ii) back pain, defined as mean of total back pain and nocturnal back pain; (iii) patient's global assessment; and (iiii) the physical function, defined as BASFI. The categories contributing to the PedACR 30 Score are the physician's global assessment of subject's disease activity (numeric rating scale, NRS), the parents' global assessment of subject's overall well-being (NRS), number of active joints (swelling not due to deformity or in joints without swelling, limitation of motion (LOM) plus pain and/or tenderness), number of joints with LOM, Childhood Health Assessment Questionnaire (CHAQ) and C-reactive protein (CRP). In addition, the erythrocyte sedimentation rate (ESR) was recorded.

The primary endpoint was the achievement of ASAS40 at week 12. A responder has been defined as a patient who achieved improvement in at least three of four domains contributing to the ASAS, with no worsening in the remaining domain. An improvement of a single domain is defined as a decrease of ≥40% and ≥2 points on the rating scale ranging from 0 to 10. Worsening of a single domain is defined as increase of >20% or >1 point on the rating scale.

The PedACR30 and the PedACR70 scores were calculated as published [32].

### Statistical analysis

The efficacy analyses were performed in the intention-to-treat (ITT) population. The ITT population was defined as all subjects who were randomized and who have received at least one dose of the drug and at least one post-dose efficacy assessment at any dose. Further, to address the possible impact of major protocol violations, an additional 'per-protocol' population was defined excluding all subjects with premature discontinuation or major protocol violations. No substantial differences were observed between the ITT and the per protocol analysis populations. Therefore, the ITT analysis was chosen. Data on patients who prematurely discontinued were included in a last observation carried forward modus. The efficacy variables were analyzed either using Pearson's χ2 test, U-test, independent t-tests, analysis of covariance (ANCOVA) for repeated measures.

### Independent ethical committee

The study was conducted in accordance with the protocol ICH GCP, FDA regulations governing clinical study conduct, ethical principles that have their origin in the Declaration of Helsinki, 1996 revision and 2000 revision with subsequent clarifications, and all applicable local regulations. Before the study was initiated, the study protocol, the Informed Consent Form and Subject Information were submitted to the responsible independent ethics committee of the Aerztekammer Nordrhein, Duesseldorf, Germany for review and it was approved on 02 May 2008. Parents/legal guardian signed the Informed Consent form before any study-related procedures occurred.

## Results

### Patient population

**Figure 1:**
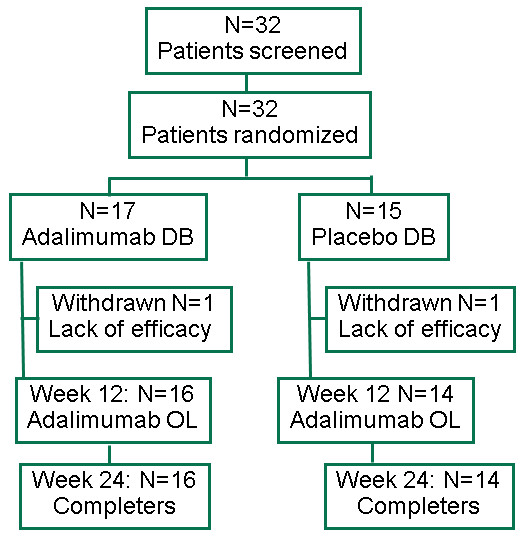
**Patient disposition**. Flow chart showing the disposition of the study patients from initial screening through week 24. All patients screened have been randomized. One patient each in both groups discontinued prematurely (before week 4) due to lack of efficacy. All patients reaching week 12 of the placebo controlled phase of the study have been admitted to the open label phase. There were no further drop outs.

The target population of 50 patients could not be reached and recruiting was stopped prematurely because of the expiration date of the study drug. Seventeen patients were randomized to receive adalimumab 40 mg/2 wks and 15 patients received placebo. Two patients (one of each group) discontinued prematurely due to lack of efficacy and were labeled as non-responders (Figure 1). Sixteen patients of the adalimumab group and 14 patients of the placebo group remained in the study and reached week 12, the final end point of the controlled part of the study. All these 30 patients entered into the open labeled phase at week 12 and remained in the study till week 24. One patient in the adalimumab group has been diagnosed with Complex Pain Syndrome and turned out to be an ASAS non-responder in both phase 1 and phase 2 of the study.

The detailed baseline patients' characteristics are outlined in Table 1. The median duration of specific complaints was 34.9 months, the median duration since diagnosis was 6.5 months. HLA-B27 was present in 76% of cases. AS in the family was known in 37.5% of patients. Two to six (median 2.5) different previous AS medications were reported; these were, predominantly, conventional NSAIDs (100.0%), COX-2-inhibitoirs (28.1%), oral corticosteroids (40.6%), intra-articular corticosteroids (37.5%), sulfasalazine (34.4%), methotrexate (31.3%) and COX-2-inhibitors (28.1%). Comparability of study groups was analyzed thoroughly at baseline. A higher proportion of patients of the placebo cohort had received oral corticosteroids (U-test, *P *= 0.04).

**Table 1:** Patient's characteristics

	Adalimumab n = 17	Placebo n = 15
Age (years; Mean +/-SD)	15.1 +/- 1.5	15.5 +/- 1.7

Gender female (%)	7 (41%)	8 (53%)

Duration since diagnosis (years; Mean +/- SD)	0.9 +/- 1.1	2.5 +/- 2.8

Duration since first symptoms (years; Mean +/-SD)	2.3 +/- 1.5	4.0 +/- 3.1

Family history with ankylosing spondylitis	6 (35.3%)	6 (40.0%)

HLA B27 positive	10/14 (71.4%)	9/11 (81.8%)

Previous treatment courses		
Conventional NSAIDs	17 (100%)	15 (100%)
Coxibes	5 (29.4)	4 (26.7)
Corticosteroids, oral Corticosteorids, intraarticular	4 (23.5) 6 (35.3)	9 (60%) # 6 (40)
Methotrexate	4 (23.5)	6 (40)
Sulfasalazine	4 (23.5)	7 (46.7)

Concomitant medication		
Conventional NSAIDs	14 (82.4%)	14 (93.3%)
Coxibes	1 (5.9%)	
Corticosteroids, oral	3 (17.6%)	5 (33.3%)

No. of active joints mean (SD)	3.8 (2.6)	4.9 (5.1)

No. of joints with LOM mean (SD)	2.2 (2.6)	4.7 (5.9)

BASDAI Spinal inflammation mean (SD)	4.29 (1.97)	5.24 (2.34)

BASDAI Total score mean (SD)	4.75 (1.30)	5.45 (1.50)

Back pain at any time mean (SD)	6.0 (1.4)	7.2 (1.6)*

Back pain at night mean (SD)	5.3 (2.4)	4.9 (2.6)

Pain score mean (SD)	5.65 (1.58)	6.07 (1.46)

Patient's Global NRS mean (SD)	5.94 (1.85)	6.87 (1.88)

Physical function (BASFI) mean (SD)	3.85 (1.80)	4.17 (2.31)

Physical function (CHAQ) mean (SD)	0.993 (0.420)	0.958 (0.397)

Physician's Global NRS mean (SD)	5.59 (1.77)	6.07 (2.19)

Parent's Global NRS mean (SD)	5.94 (1.39)	6.07 (2.19)

Back pain at any time mean (SD)	6.0 (1.4)	5.60 (2.06)

#A higher proportion of patients of the placebo cohort had received oral corticosteroids (U-test, *P *= 0.04). *Back pain at any time was the only significantly different clinical item between both treatment groups.

### Efficacy results

In the double-blind part of the study, more patients on adalimumab achieved an ASAS40, the primary outcome parameter, at week 4 (41%), week 8 (53%) and week 12 (53%) than on placebo (20%, 33%, 33%), while differences at week 8 only reached the border significance (*P *= 0.05). Secondary outcome parameter, ASAS20 and PedACR30 and -70 response rates at 4, 8 and 12 weeks were higher in the adalimumab group (Table 2).

**Table 2:** Efficacy: primary and secondary endpoints

Endpoint	Number (%)		Odd's ratio (95% CI)	*P*-value*
			
	Adalimumab (n = 17)	Placebo (n = 15)		
**Primary endpoint**				

ASAS40 week 4	7 (41)	3 (20)	2.3 (0.6 to 13.8)	0.20
week 8	9 (53)	3 (20)	3.6 (0.9 to 21.9)*	0.05
week 12	9 (53)	5 (33)	1.6 (0.5 to 9.5)	0.26

**Secondary endpoint**				

ASAS20 week 4	9 (53)	10 (59)	4 (27)	4 (27)
week 8	2.4 (0.7 to 13.7)	3.0 (0.9 to 17.6)	0.13	0.07
week 12	9 (53)	5 (33)	1.6 (0.5 to 9.5)	0.26

PedACR30 week 4	9 (53)	4 (27)	2.4 (0.7 to 13.7)	0.13
week 8	13 (76)	5 (33)	5.1 (1.4 to 30.7)	0.01
week 12	11 (65)	6 (40)	2.0 (0.7 to 11.5)	0.16

PedACR70 week 4	5 (29)	1 (6.7)	6.8 (0.6 to 57.1)	0.10
week 8	7 (41)	2 (13)	4.1 (0.8 to 26.8)	0.08
week 12	9 (53)	4 (27)	2.4 (0.7 to 13.7)	0.13

In this intention to treat analysis one patient of each group who discontinued prematurely was labeled as non-responder. * Chi square test.

In the adalimumab group at week 12, a decrease of all single disease activity parameters was noted compared to baseline. The mean (+/-SD) BASDAI spinal inflammation score decreased from 4.3 ± 2.1 to 1.5 ± 1.7 (-66%; *P *<0.001), the back pain score decreased from 5.5 ± 1.6 to 2.8 ± 2.9, (-48%; *P *<0.005), the BASFI score decreased from 3.8 ± 1.8 to 2.0 ± 2.3 (-47%; *P *<0.02), the CHAQ-DI score improved from 1.0 ± 0.4 to 0.6 ± 0.7 (-65%; *P *<0.005), the ESR improved from 23 +/- 27 mm/h to 6 +/- 3 mm/h (- 75%; *P *<0.05) and finally the CRP improved from 13 +/- 22 mg/l to 4 +/- 0.8 mg/l (- 80%; *P *= 0.07).

In the placebo group as well, some disease activity parameters improved while only the back pain score decreased significantly from 6.2 ± 1.4 to 4.3 ± 2.4 (-31%; *P *<0.02). In the placebo group the mean (+/- SD) BASDAI spinal inflammation score decreased from 5.0 ± 2.2 to 3.8 ± 3.0 (-25%; n.s.), the BASFI score decreased from 4.2 ± 2.3 to 3.3 ± 2.6 (-20%; n.s.), the Childhood Health Assessment Questionnaire-Disability Index (CHAQ-DI) score improved from 1.1 ± 0.8 to 0.8 ± 0.6 (-31%; n.s.), the ESR increased from 16 +/- 16 mm/h to 18 +/- 20 mm/h (+ 7%, n.s.) and finally the CRP increased from 5.4 +/- 9.6 mg/l to 10 +/- 26 mg/l (+ 88%) at week 12.

**Figure 2:**
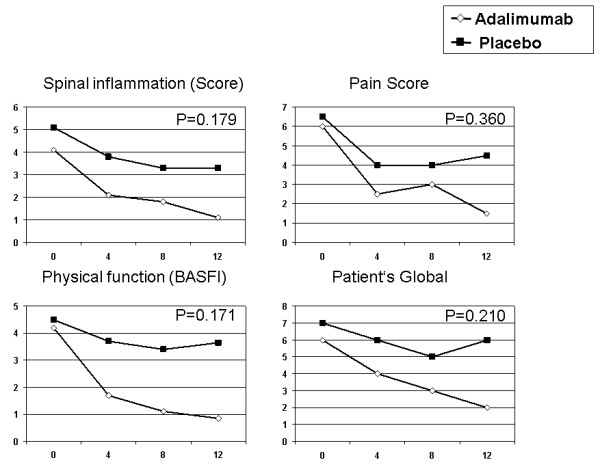
**Reduction of the Items contributing to the ASAS-Score during 12 weeks of blinded treatment**. (**A**) Spinal inflammation Score, (**B**) Pain Score, (**C**) Physical function (Bath ankylosing spondylitis functional index), (**D**) Patient's Global Assessment of disease activity. The median is given.

Improvement of the median of the individual disease activity parameters composing the ASAS score is given in Figure 2. The reduction of the "Spinal inflammation Score", the "Pain Score", the "Physical function score" (BASFI) and the "Patient's Global Disease Activity Score" was more pronounced in patients of the adalimumab group than the placebo group, but did not reach significance in the intergroup comparison.

**Figure 3:**
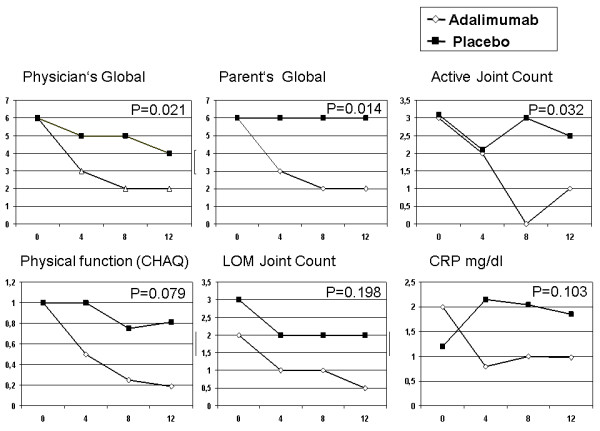
**Clinical improvement of the Items contributing to the PedACR during 12 weeks of blinded treatment**. (**A**) Physician's Global Assessment of disease activitry, (**B**) Parent's Global Assessment of overall well being, (**C**) Numbers of joints with active arthritis, (**D**) Childhood Health Assessment Questionnaire Disability Index, (**E**) joints with limited range of motion and serum levels of C-reactive Protein. The median is given. The global treatment difference was significant for physician's global assessment (*P *= 0.021), parents' global assessment (*P *= 0.016). At week 8 significant differences were noted for physician's global assessment (*P *= 0.006), parents' global assessment (*P *= 0.025), the number of active joints (*P *= 0.0007), the CHAQ-DI (*P *= 0.017).

Improvement of the individual disease activity parameters composing the PedACR Score is given in Figure 3. In patients of the adalimumab group, a marked reduction of the median of the "Physician's Global Disease Activity Score", the "Parent's Global Assessment of subject's overall well-being", the "Active Joint Count", the "Physical Function Score" (CHAQ), the "LOM Joint Count" and the CRP are evident, while there was no change in patients of the placebo group for "parent's global" and "active joint count", a weak decrease only for "physician's global", "CHAQ" and "limited motion joint count" or an increase for the CRP level.

Although a randomization has been performed, there were some differences between the patient groups at baseline (Table 1). Therefore, for statistical comparison, baseline adjustment has been performed (Table 3). The course of all single items was analyzed at different time points and in the repeated measurement design to assess change over time in the ITT population. The global treatment difference is outlined in Table 3. The difference for physician's global assessment of subject's disease activity after 8 weeks (*P *= 0.014) and 12 weeks (*P *= 0.043) were statistically significant, while the tests after 4 weeks (*P *= 0.067) showed marked tendencies in favor of adalimumab. The course of parents' global assessment of patient's overall well-being showed significant differences (week 4: *P *= 0.022, week 8: *P *= 0.045, week 12: *P *= 0.013) in the entire course of double-blind treatment phase. The course of the number of active joints showed significant advantages of adalimumab at week 8 (*P *= 0.002).

**Table 3:** Comparison of PedACR and ASAS domains after baseline adjustment

		Week 4	Week 8	Week 12	Treatment *P-*value*
Physicians' Global	Adalimumab	2.97 +/- 0,53	2.44 +/- 0.53	2.67 +/- 0.54	
5.81#	Placebo	4.43 +/- 0.57	4.43 +/- 0.57	4.31 +/- 0.57	
	Difference (95% confidence Interval)	-1.47 +/- 0.78	-2.00 +/- 0.78	-1.64 +/- 0.79	0.021

Parents' Global	Adalimumab	3.45 +/- 0.62	2.86 +/- 0.62	2.85 +/- 0.63	
5.78	Placebo	5.62 +/- 0.66	4.75 +/- 0.66	5.24 +/- 0.67	
	Difference (95% confidence Interval)	-2.17 +/- 0.91	-1.89 +/- 0.91	-2.39 +/- 0.93	0.014

No. Active Joints	Adalimumab	1.72 +/- 0.49	1.31 +/- 0.49	1.99 +/- 0.5	
4.31	Placebo	2.59 +/- 0.52	3.65 +/- 0.52	2.83 +/- 0.53	
	Difference (95% confidence Interval)	-0.87 +/- 0.72	-2.35 +/- 0.72	-0.84 +/- 0.73	0.032

No. LOM Joints	Adalimumab	1.60 +/- 0.47	1.78 +/- 0.47	1.84 +/- 0.48	
3.34	Placebo	2.38 +/- 0.50	2.65 +/- 0.50	2.01 +/- 0.52	
	Difference (95% confidence Interval)	-0.78 +/- 0.69	-0.87 +/- 0.69	-0.17 +/- 0.71	0.198

CHAQ	Adalimumab	0.57 +/- 0.12	0.42 +/- 0.12	0.44 +/- 0.12	
0.98	Placebo	0.80 +/- 0.12	0.75 +/- 0,12	0.74 +/- 0.13	
	Difference (95% confidence Interval)	-0.22 +/- 0.17	-0.34 +/- 0.17	-0.30 +/- 0.17	0.079

CRP	Adalimumab	0.99 +/- 2.77	3.92 +/- 2.77	1.98 +/- 2.89	
10.34	Placebo	5.91 +/- 3.05	8.69 +/- 3.05	9.72 +/- 3.05	
	Difference (95% confidence Interval)	-4.92 +/- 4.14	-4.76 +/- 4.14	-7.74 +/- 4.23	0.103

Spinal inflammation	Adalimumab	2.60 +/- 0.56	2.64 +/- 0.56	2.05 +/- 0.56	
4.73	Placebo	3.56 +/- 0.60	3.51 +/- 0.60	3.50 +/- 0.60	
	Difference (95% confidence Interval)	-0.97 +/- 0.83	-0.87 +/- 0.83	-1.45 +/- 0.83	0.179

Pain Score	Adalimumab	3.60 +/- 0.59	3.60 +/- 0.59	3.23 +/- 0.60	
5.84	Placebo	4.38 +/- 0.63	3.98 +/- 0.63	4.33 +/- 0.64	
	Difference (95% confidence Interval)	-0.78 +/- 0.87	-0.38 +/- 0.87	-1.11 +/- 0.88	0.360

Patients' Global	Adalimumab	4.50 +/- 0.54	3.97 +/- 0.54	3.76 +/- 0.55	
6.38	Placebo	5.44 +/- 0.58	4.70 +/- 0.58	4.82 +/- 0.59	
	Difference (95% confidence Interval)	-0.94 +/- 0.80	-0.74 +/- 0.80	-1.06 +/- 0.81	0.210

BASFI	Adalimumab	2.53 +/- 0.47	2.38 +/- 0.47	2.14 +/- 0.48	
4.00	Placebo	3.36 +/- 0.50	3.28 +/- 0.50	3.09 +/- 0.51	
	Difference (95% confidence Interval)	-0.84 +/- 0.69	-0.90 +/- 0.69	-0.95 +/- 0.70	0.171

*Total treatment difference, repeated measurement design. # Baseline adjusted value. $ ANCOVA analysis.

The course of CHAQ showed nearly significant differences in favor of adalimumab at week 8 (*P *= 0.054).

**Figure 4:**
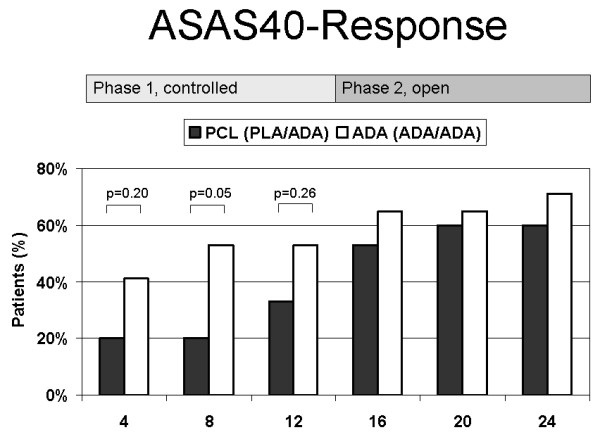
**ASAS40**. Percentage of patients who achieved an ASAS40. *P*-value of adalimumab treated patients versus placebo is outlined based on an intention-to-treat analysis (χ2-test). One patient in each group discontinued prematurely before week 4. The data were included in a last observation carried forward modus. At week 12, all patients remaining patients switched to open label adalimumab.

In the open label phase of the study, all patients received active treatment with adalimumab. The improvements obtained in the adalimumab group were maintained or even augmented in the open-label follow-up study phase in those patients of the sequence adalimumab - adalimumab (Figure 4A-D). Due to the improvement reached in the patients treated by the sequence placebo-adalimumab in the open label study phase, the treatment differences observed after 12 weeks between placebo and adalimumab showed decreasing tendencies. After 24 weeks, there were no or only slight differences in favor of the long-term treatment with adalimumab.

### Safety results

In the double-blind study phase 27 adverse events were observed in 10 of 17 patients on adalimumab (58.8%) and 29 adverse events were observed in 10 of 15 patients on placebo (66.7%), predominantly as 'Infections and infestations' (adalimumab: N = 6; placebo: N = 6) and 'General disorders and administration site conditions' (adalimumab N = 3; placebo: N = 4). Adverse events at least possibly related to the study medication (adverse drug reactions) were reported in four patients on adalimumab (23.6%) and five patients on placebo (33.3%), predominantly as 'General disorders and administration site conditions' (adalimumab: N = 3; placebo: N = 3). Drug related infections were reported only once on adalimumab (appendicitis).

In the open study phase, there were 19 adverse events in 10 of 16 patients of the sequence adalimumab-adalimumab (62.5%) and 29 adverse events in 7 of 14 patients in the sequence placebo-adalimumab (50.0%). The most common events were 'Infections and infestations' (adalimumab-adalimumab: N = 8; placebo-adalimumab: N = 6). Deviating from the double-blind study phase, the infections were predominantly classified by the local investigators as related to the study medication (N = 9), while 'General disorders and administration site conditions' occurred in only N = 3 patients.

Serious adverse events occurred in three patients during the double-blind study phase, two on adalimumab (appendicitis, tendonitis) and one on placebo (gastritis) and in four patients during the open study phase upon adalimumab, two in the sequence adalimumab-adalimumab (vertigo, general pain) and two in the sequence placebo-adalimumab (colitis, pyelonephritis). In two patients the events (appendicitis and pyelonephritis) were possibly related to adalimumab, the further serious adverse events were not related or probably not related, respectively, to the study medication (Table 4). No clinically significant laboratory abnormalities were related to the treatment with adalimumab. In all, the tolerability of the study medication was assessed very good or good in the majority of patients (patient's overall assessment: 27/32 (84.4%), investigator's overall assessment: 30/32 (93.8%)).

**Table 4:** Serious adverse events

Adalimumab	Placebo
Appendicitis^1,4^	Gastritis^2,4^
Tendonitis^2,4^	
Sequence Adalimumab-Adalimumab	Sequence Placebo- Adalimumab
Pain^2,4^	Colitis^3,4^
Vertigo^3,4^	Pyelonephritis^1,4^

^1^Judged as possibly related; ^2^Judged as not related; ^3^Judged as probably not related; ^4^Outcome: recovered completely. There were no events of malignancy, demyelinating disorders or deaths.

## Discussion

This randomized, double-blind trial showed the clinical efficacy of the TNF inhibitor adalimumab in patients with JoAS. Treatment with adalimumab for 12 weeks was associated with a reduction in disease activity as assessed by a number of clinical end points, biochemical markers of disease, and quality of life. These data show that TNF antagonism is a valid approach to the short term treatment of juvenile ankylosing spondylitis.

Improvement occurred rapidly, mostly within four to eight weeks. The strength of improvement, furthermore, increased from week 12 to week 24 in those patients already treated with adalimumab in phase 1 of the study. Patients receiving placebo in phase 1 of the study also demonstrated marked improvement in phase 2 after they were switched from placebo to adalimumab.

The comparison of the defined primary objective ASAS40 at 12 weeks failed to demonstrate statistically significant superiority. Due to the impressive differences outlined in Tables 2 and 3 and in Figures 2, 3 and 4, this failure is most likely due to the small sample sizes. Unfortunately, recruitment had to be stopped before the target population of 50 patients could be reached. Furthermore, two patients (one of each group) discontinued prematurely and assessment of the efficacy of a 12 weeks trial of the study drug, therefore, was impossible. One patient turned out to be classified with pain amplification syndrome and failed to meet the improvement criteria.

Reiff *et al*. presented a small open study using etanercept for treatment of JoAS [34]. Eight patients (seven males), with a mean age of 15.9 years (range 12 to 25 years) suffering from juvenile ankylosing spondylitis for a mean of 4.5 years (range 1.2 to 17.5 years) were included. Six patients were HLA-B27 positive. Treatment has been performed with etanercept at an average dosage of 0.4 mg/kg body weight, which is the dosage recommended for treatment of polyarticular JIA [25]. The therapeutic effects were evident for up to more than 24 months. In another open study on 10 patients with juvenile spondylarthritis, improvement with anti-TNFα therapy has been demonstrated using either infliximab (n = 8) or etanercept (n = 2). [35].

A 12-week, randomized, double-blind, placebo-controlled trial using infliximab showing efficacy and safety of TNF-inhibition in juvenile-onset spondyloarthritis trial was presented as abstract only [36]. In this study, the American College of Rheumatology pediatric core criteria definition of improvement in juvenile arthritis, which currently have not been validated for juvenile spondylarthropathies, have been used only. Dramatic improvement could be shown in this study. In our controlled trial, in addition to the ACR criteria, we also applied the ASAS Working Group response criteria ASAS20 and ASAS40, although they have not so far been validated for juvenile AS. Improvements were shown with both sets of criteria, the PedACR and the ASAS criteria.

Adalimumab was well tolerated in this study. Only one patient terminated the treatment prematurely, (partly) due to adverse events (disease flare with trochanter enthesitis on both sides). Serious adverse events were observed in seven patients. In two patients the respective events (appendicitis, pyelonephritis) were possibly related to adalimumab. Clinical significant laboratory abnormalities related to adalimumab were not detected.

In the majority of randomized controlled trials in childhood rheumatic diseases, withdrawal study designs were used, but there remain questions of efficacy as well as of safety. Therefore, for the present study, a primary placebo-controlled design was chosen. Here, patients treated with adalimumab showed a continuous improvement with an increasing intensity with ongoing treatment. The strength of improvement also increased during the open label period from week 12 to week 24. Superiority over those patients treated with placebo was especially evident at week 4 and week 8. These differences were statistically significant although the total patient number included in the study was small. However, at week 12 the manifestations in some of the placebo patients improved, resulting in a loss of significance of the inter-group differences. In these patients improvement may be due to an effect of concomitant treatment with non-steroidal anti-inflammatory drugs, although their dosage was stable before the patients participated to the study, or was explained by a spontaneous remitting course. This effect, however, also may be in part responsible for the increasing improvement in all patients with prolonged treatment.

Except for non-steroidal anti-inflammatory drugs, so far there is no established alternative treatment option for patients with axial JoAS. Sulfasalazine showed some efficacy in treatment of JIA, especially in HLA B27-associated arthritis [37]. In a clinical trial on polyarticular JIA, the effect on joint tenderness, joint swelling, joint score and laboratory parameters was only marginally significant [38]. In patients with juvenile spondyloarthritis, a placebo-controlled double-blind study demonstrated an advantage of sulfasalazine over placebo in peripheral joint involvement [39]. There is no study on sulfasalazine in juvenile patients who have been classified as ankylosing spondylitis, but according to studies in adult AS, sulfasalazine probably has no effect on axial involvement [40,41].

For this study, modified NY criteria by requiring MRI rather than radiography was used due to (1) ethical issues involved with radiation and (2) newer concepts of classification in which MRI is sufficient for diagnosis of axial spondyloarthritis. In this study none of the 34 patients with radiographic sacrioiliitis had current back pain [42].

TNF-antagonists open new perspectives for treatment of juvenile spondylarthritis and, especially, juvenile ankylosing spondylitis since they have effected dramatic improvements also in patients with severe, and so far intractable, disease. Furthermore, the velocity and the strength of its effects on clinical activity are remarkable.

With this double-blind controlled trial we offer data on the efficacy and, although experienced on a limited number of patients, on safety of adalimumab in children with juvenile ankylosing spondylitis.

### Limitations

Shortcomings of the study are the low number of patients attributed to the rarity of the disease and the differences between both the adalimumab patient group and the placebo patient group at baseline, despite randomization. Furthermore, it would have been interesting to have MRI data at week 12 and 24 in the patients; perhaps we would have seen more differences between the groups.

## Conclusions

This study, performed in compliance with ICH Good Clinical Practice, showed a significant superiority of adalimumab compared with placebo in the treatment of juvenile ankylosing spondylitis. The superior efficacy was especially based on numerical rating scales, laboratory measures of inflammation and questionnaires for functional disorders. Adalimumab was well tolerated and highly effective in the treatment of juvenile ankylosing spondylitis in children and adolescents aged 12 to <18 years and should be considered in the treatment of JoAS that is active and refractory to NSAID.

## Abbreviations

ADA: Adalimumab; AE: Adverse event; ANCOVA: analysis of covariance; AS: ankylosing spondylitis; ASAS: Assessment of SpondyloArthritis international Society; BASDAI: Bath Ankylosing Spondylitis Disease activity score; BASFI: Bath Ankylosing Spondylitis Functional Index; CHAQ-DI: Childhood Health Assessment Questionnaire-Disability Index; CRP: C-Reactive Protein; DMARD: Disease modifying drug; ESR: erythrocyte sedimentation rate; FDA: Food and Drug Administration; ICH GCP: International Conference on Harmonisation Good Clinical Practice; ITT: intention-to-treat; JIA: juvenile idiopathic arthritis; JoAS: juvenile onset ankylosing spondylitis; LOM: limitation of motion; MRI: Magnet resonance imaging; NRS: numeric rating scale; NSAID: Nonsteroidal anti-inflammatory drug; PedACR criteria: pediatric version of the American Colleague or rheumatology criteria; PLC: Placebo; SAE: Serious adverse event; TNF: tumor necrosis factor

## Competing interests

GH declared funding for clinical trials, advisory board membership and the receipt of honorary fees from Abbott, Pfizer, Novartis, Roche/Chugai, Genzyme, UCB, Bristol-Myers Squibb. H-IH declared advisory board membership and speaking fees from Abbott, Pfizer, Roche/Chugai and Swedish Orphan. IF declared advisory board membership for Abbott, Pfizer, Novartis and Chugai. JK-D declared consulting fees and honoraries from Novartis. GG declared advisory board membership of Abbott. KM declared research grants, honoraria and consulting fees from Abbott, Bristol-Myers Squibb, Chugai, Medac, Novartis, Pfizer and Roche. SF, NT, AT, MB and RT declared no competing interests.

## Authors' contributions

The study was designed by GH. Patients were included and data were collected by all the investigators. All authors decided to submit this manuscript, and all vouch for the veracity of the data, analyses and trial protocol. GH wrote the first draft of this manuscript. Subsequent writing assistance was provided by HIH. All authors have read and approved the manuscript for publication.
